# Microarray data mining using landmark gene-guided clustering

**DOI:** 10.1186/1471-2105-9-92

**Published:** 2008-02-11

**Authors:** Pankaj Chopra, Jaewoo Kang, Jiong Yang, HyungJun Cho, Heenam Stanley Kim, Min-Goo Lee

**Affiliations:** 1 Dept. of Computer Science, North Carolina State Universtiy, Raleigh, NC-27606, USA; 2 Dept. of Computer Science and Engineering, Korea University, Seoul, Korea; 3 Dept. of Biostatistics, College of Medicine, Korea University, Seoul, Korea; 4 Case Western Reserve University, Cleveland, OH-44106, USA; 5 Dept. of Statistics, Korea University, Seoul, Korea; 6 Bioinformatics and Functional Genomics Laboratory, Graduate School of Medicine, Korea University, Seoul, Korea; 7 Department of Physiology, College of Medicine, Korea University, Seoul, Korea

## Abstract

**Background:**

Clustering is a popular data exploration technique widely used in microarray data analysis. Most conventional clustering algorithms, however, generate only one set of clusters independent of the biological context of the analysis. This is often inadequate to explore data from different biological perspectives and gain new insights. We propose a new clustering model that can generate multiple versions of different clusters from a single dataset, each of which highlights a different aspect of the given dataset.

**Results:**

By applying our SigCalc algorithm to three yeast Saccharomyces cerevisiae datasets we show two results. First, we show that different sets of clusters can be generated from the same dataset using different sets of landmark genes. Each set of clusters groups genes differently and reveals new biological associations between genes that were not apparent from clustering the original microarray expression data. Second, we show that many of these new found biological associations are common across datasets. These results also provide strong evidence of a link between the choice of landmark genes and the new biological associations found in gene clusters.

**Conclusion:**

We have used the SigCalc algorithm to project the microarray data onto a completely new subspace whose co-ordinates are genes (called landmark genes), known to belong to a Biological Process. The projected space is not a true vector space in mathematical terms. However, we use the term subspace to refer to one of virtually infinite numbers of projected spaces that our proposed method can produce. By changing the biological process and thus the landmark genes, we can change this subspace. We have shown how clustering on this subspace reveals new, biologically meaningful clusters which were not evident in the clusters generated by conventional methods. The R scripts (source code) are freely available under the GPL license. The source code is available [see Additional File 1] as additional material, and the latest version can be obtained at . The code is under active development to incorporate new clustering methods and analysis.

## Background

Microarrays have enabled scientists to monitor the activities of thousands of genes simultaneously. Clustering methods provide a useful technique for exploratory analysis of microarray data since they group genes with similar expression patterns together. It is believed that genes that display similar expression patterns are often involved in similar functions. Various clustering techniques have been proposed [1,2]. Some of the popular techniques for clustering genes employ k-means [3], hierarchical clustering [4], self-organizing maps [5] or some of their variants. Although clustering is a data exploration tool, there is a shortage of clustering algorithms that enable the exploration of a dataset from multiple different biological perspectives. Most of these conventional clustering algorithms generate only one set of clusters, thus forcing a very restricted view of gene associations. They leave little room for data exploration and re-interpretation of existing data. It would be difficult to interpret the complex biological regulatory mechanisms and genetic interactions from this restrictive interpretation of microarray expression data. In this paper we show that biologically meaningful gene clusters can be developed with our gene signature algorithm *SigCalc*. Our algorithm uses elements of subspace projection, along with existing knowledge on gene associations to come up with multiple new cluster sets. We show that each of these new cluster sets reveal biological associations that were not apparent from clustering the original gene expression data. The proposed method is fundamentally different from the conventional subspace clustering methods in that it projects the original expression data into a different information space where genes are described in relative terms against a chosen subset of genes called landmarks.

### Random Projection

Random projection is one of the dimensionality reduction techniques that is useful for eliminating features that may be irrelevant. The high dimensionality data is projected onto a smaller random subspace. Random projections and subspaces have been extensively used in data mining. They have been used to reduce dimensionality and search for similarity in clustering [6,7] and for information retrieval [8]. Some of the application areas include classification [9], image processing [10], and other machine learning topics [11,12]. The key difference between our method and other random projection methods is that we project our data onto a known set of genes that are functionally related, whereas in other methods, random points are chosen for the subspace.

### Subspace Clustering

Subspace clustering or biclustering [13,14], has been a popular method for analyzing microarray datasets. The main idea of subspace clustering is to find a subset of genes and a subset of conditions under which these genes exhibit a similar trend. The major differences between the subspace clustering and the method proposed in this paper are: (1) The subspace clusters are static; whereas, our framework provides a tool for users to choose landmark genes, and then to analyze the dataset based on these landmark genes. (2) Unlike the subspace clusters, the clusters generated from our method using the same landmarks are comparable across different datasets.

### Semi-supervised Clustering

Semi-supervised clustering [15-17] uses existing domain knowledge to guide the clustering process. One popular method is constraint based clustering, where pairwise constraints (i.e 'must-link' and 'cannot-link' pairs) guide the clustering. The objective function of the underlying clustering algorithm is modified to accomodate these constraints. Our method differs from this clustering method as it does not constrain all the landmark genes to belong to one cluster. In our biological context, it is not unusual for genes to have more than one function.

### Gene Ontology

Gene Ontology (GO) is a collection of controlled vocabularies that describe the biology of a gene product [18]. It consists of approximately 20,000 terms arranged in three independent ontologies: Biological Process, Cellular Component, and Molecular function, each represented by a directed acyclic graph (DAG). Gene Ontology has proven to be very important for secondary analysis of microarray expression data [19], and a wide range of tools have been developed to aid in this analysis. A comprehensive analysis of the available tools is given by Khatri [20]. Some of the prominent ones are ontoTools [21], GOminer [22], and GOstat [23].

In this paper we use the Biological Process ontology. A Biological Process (BP) is defined as "A phenomenon marked by changes that lead to a particular result, mediated by one or more gene products". As of 2006, there were approximately 10,000 GO terms associated with Biological Process [24]. We use Gene Ontology to provide external validation for the clusters. We use statistical significance tests to determine if the genes in a cluster belong to a specific Biological Process. A biologically meaningful cluster would consist of many genes that are annotated to a specific *GO term*.

## Results and Discussion

### Results

**Figure 1:**
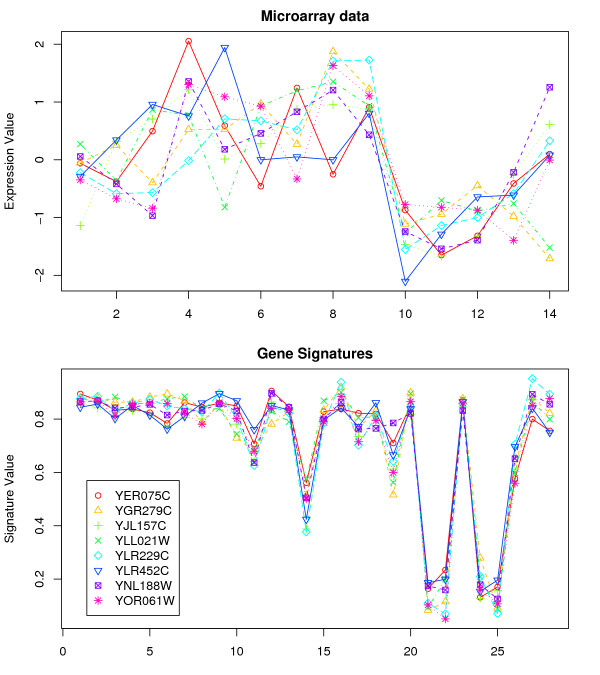
**Comparison of microarray expression data with gene signatures for genes that clustered together using gene signatures**. Gasch dataset: Genes associated with *multi-organism process *(GO:0051704) were clustered together.

**Figure 2:**
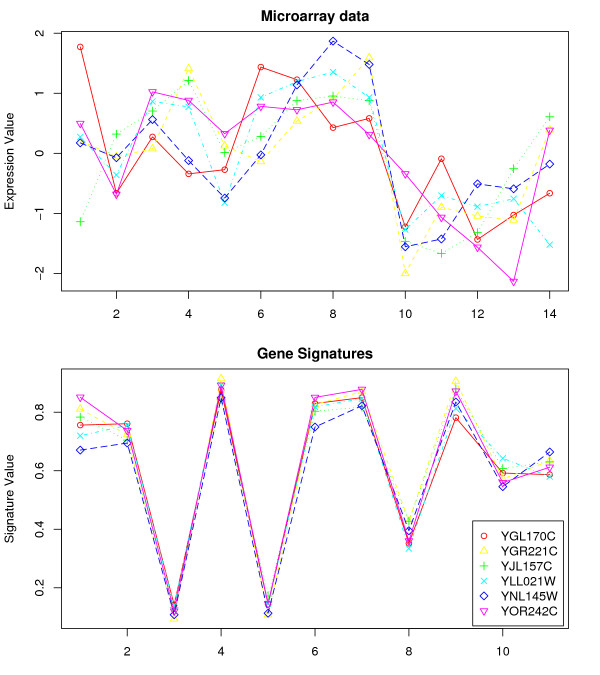
**Comparison of microarray expression data with gene signatures for genes that clustered together using gene signatures**. Gasch dataset: Genes associated with *reproduction *(GO:0000003) were clustered together.

In the gene signature model, genes are points in a projected subspace whose coordinates are the landmark genes. The gene signature consists of relative distance to these landmark genes. So, by changing the landmark genes, a different perspective of the subspace can be obtained. Even using the same clustering algorithm, we can get different sets of clusters by changing this subspace. We repeated gene signature clustering for several biological processes (i.e., we used several different sets of landmark genes). The details for the *overlapping GO terms *and the *unique GO terms*, using different biological processes as landmarks for the Spellman dataset are shown in Table 1 (see Additional File 2 for DeRisi dataset). We analyzed genes in some of the clusters that produced the *unique GO terms*. These genes, annotated to the same GO term, clustered together when gene signatures were used, but did not cluster together when the original microarray data was used. Some of these genes are shown in Figures 1 and 2.

**Table 1:** Details of overlaps between significant GO terms found by original clustering of microarray data, and those found by using gene signature clustering for the Spellman dataset.

*Biological process used for landmark genes*	*Number of Landmark Genes*	*Number of Original GO terms*	*Number of Overlapping GO terms*	*Number of Unique GO terms*
proteolysis	51	182	120	41
electron transport	20	182	126	44
regulation of transcription	100	182	126	41
protein biosynthesis	194	182	101	20
carbohydrate metabolism	121	182	142	58
signal transduction	52	182	121	53
ubiquitin-dependent protein catabolism	40	182	129	61

As illustrated, the gene expression patterns do not appear to be highly correlated, while the gene signatures show a strong correlation. For example, in the Gasch dataset, eight genes all relating to the GO term *multi-organism process *(GO:0051704), were in one cluster when gene signatures (with electron transport as landmark) were used. These genes did not cluster together with the original microarray data. Similarly, the six genes YGL170C, YGR221C, YJL157C, YLL021W, YNL145W and YOR242C, associated with *reproduction *(GO:0000003), only clustered together when gene signatures (with protein ubiquitination) were used. Although the biological significance between the landmark genes and the new GO term discovered is not immediately clear in this case, there might be some inherent relationships between them that are worth further investigation. Nonetheless, there were many other GO terms discovered using signatures (but not with the original expression data) whose associations with signature terms are much clearer. Some of these terms are investigated in detail in the discussion section.

**Figure 3:**
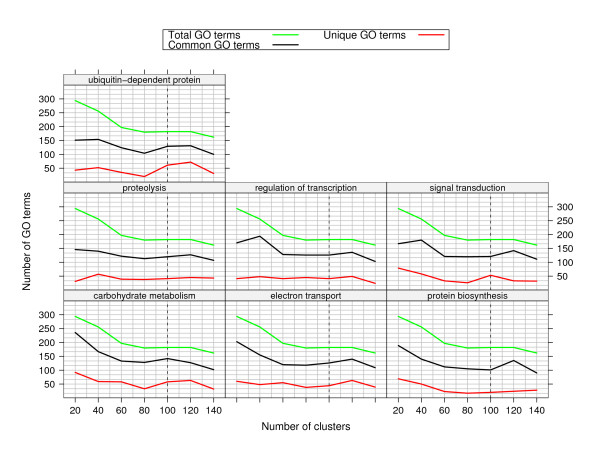
**Number of GO terms for varying number of clusters**. For each landmark, a number of unique GO terms are found irrespective of the number of clusters.

In order to test the effect of the number of clusters on the number of unique GO terms discovered for each landmark, we performed an experiment varying the numbers of clusters from 20 to 140. The results are shown in Figure 3. These indicate that there are a substantial number of unique GO terms for each set of landmark genes, that are largely independent of the number of clusters.

Next, we compared the *unique GO terms *from two datasets for different landmark genes. Table 2 shows details of this comparison for three datasets taken two at a time. For example, the first and second column indicate the number of *unique GO terms *found for the Spellman and the Gasch datasets. The third column indicates the number of *unique GO terms *that were common between the Spellman and the Gasch datasets, and the p-value associated with this. In effect, this indicates the number of significant GO terms found in both datasets, by clustering of gene signatures, that were not found in the original clustering of either of the two datasets. Similarly, Table 3 shows the comparison when SOM was used for clustering. As can be seen from the tables, both the clustering algorithms produced a substantial number of *unique GO terms *that were common across datasets.

**Table 2:** Common Unique GO Terms between datasets (taken two at a time), using Tight Clustering algorithm.

	Spellman-Gasch (2038 genes) Unique GO terms			Gasch-DeRisi (2474 genes) Unique GO terms			Spellman-DeRisi (1408 genes) Unique GO terms		
	
*Biological process used to get landmark genes*	*Spellman*	*Gasch*	*Common (p-value)*	*Gasch*	*DeRisi*	*Common (p-value)*	*Spellman*	*DeRisi*	*Common (p-value)*
proteolysis	28	89	12 (7.7 × 10^-6^)	117	80	27 (1.2 × 10^-7^)	32	17	3 (3.4 × 10^-2^)
electron transport	28	89	9 (1.3 × 10^-3^)	121	125	28 (6.5 × 10^-4^)	47	59	15 (1.6 × 10^-6^)
regulation of transcription	23	57	5 (1.3 × 10^-2^)	83	76	20 (1.6 × 10^-6^)	31	24	5 (2.8 × 10^-3^)
protein biosynthesis	32	85	7 (2.6 × 10^-2^)	101	83	20 (1.4 × 10^-4^)	21	53	1 (3.2 × 10^-1^)
carbohydrate metabolism	22	72	7 (1.4 × 10^-2^)	97	81	16 (3.6 × 10^-3^)	28	33	1 (3.6 × 10^-1^)
signal transduction	43	68	23 (1.0 × 10^-15^)	76	98	22 (1.6 × 10^-6^)	44	28	8 (1.6 × 10^-4^)
protein folding	29	72	10 (6.3 × 10^-5^)	110	81	31 (4.9 × 10^-11^)	24	32	4 (1.8 × 10^-2^)
intracellular protein transport	38	79	9 (5.1 × 10^-3^)	137	83	25 (7.6 × 10^-5^)	33	43	7 (2.4 × 10^-3^)
lipid metabolism	43	73	17 (1.3 × 10^-8^)	97	85	27 (6.4 × 10^-9^)	32	27	4 (2.6 × 10^-2^)
ribosome biogenesis	66	94	22 (4.4 × 10^-7^)	111	124	22 (1.2 × 10^-2^)	55	30	9 (2.4 × 10^-4^)

**Table 3:** Common Unique GO Terms between datasets (taken two at a time), using SOM algorithm.

	Spellman-Gasch (2038 genes) Unique GO terms			Gasch-DeRisi (2474 genes) Unique GO terms			Spellman-DeRisi (1408 genes) Unique GO terms		
	
*Biological process used to get landmark genes*	*Spellman*	*Gasch*	*Common (p-value)*	*Gasch*	*DeRisi*	*Common (p-value)*	*Spellman*	*DeRisi*	*Common (p-value)*
proteolysis	28	90	1 (1.3 × 10^-1^)	90	56	17 (4.4 × 10^-6^)	29	49	8 (4.6 × 10^-4^)
electron transport	55	76	23 (1.1 × 10^-11^)	97	52	17 (4.13 × 10^-6^)	36	57	3 (2.4 × 10^-1^)
regulation of transcription	39	79	15 (5.0 × 10^-7^)	69	45	8 (9.8 × 10^-3^)	32	40	1 (2.9 × 10^-1^)
protein biosynthesis	64	71	19 (2.07 × 10^-7^)	77	69	10 (2.8 × 10^-2^)	20	72	2 (2.9 × 10^-1^)
carbohydrate metabolism	37	73	11 (1.3 × 10^-4^)	76	60	13 (4.2 × 10^-4^)	42	45	2 (2.5 × 10^-1^)
signal transduction	74	74	14 (2.5 × 10^-3^)	92	61	10 (3.7 × 10^-2^)	45	36	6 (1.9 × 10^-2^)
protein folding	47	71	5 (1.7 × 10^-1^)	77	44	5 (1.4 × 10^-1^)	39	36	4 (9.6 × 10^-2^)
intracellular protein transport	41	98	16 (3.5 × 10^-6^)	113	51	18 (6.0 × 10^-6^)	42	46	6 (1.4 × 10^-2^)
lipid metabolism	47	83	9 (2.3 × 10^-2^)	84	64	12 (5.5 × 10^-3^)	73	37	0 (1.3 × 10^-2^)
ribosome biogenesis	40	71	19 (1.6 × 10^-11^)	99	77	9 (1.4 × 10^-1^)	27	55	0 (9.7 × 10^-2^)

We also compared our gene signature model against a base line approach built using a *k*-nn classifier. We used ten fold cross validation to impute functional annotations using k-nn and clusters obtained from our model. For all the landmarks tested, our approach produced a higher classification accuracy than the *k*-nn based approach, irrespective of '*k*' (see Additional File 2).

**Figure 4:**
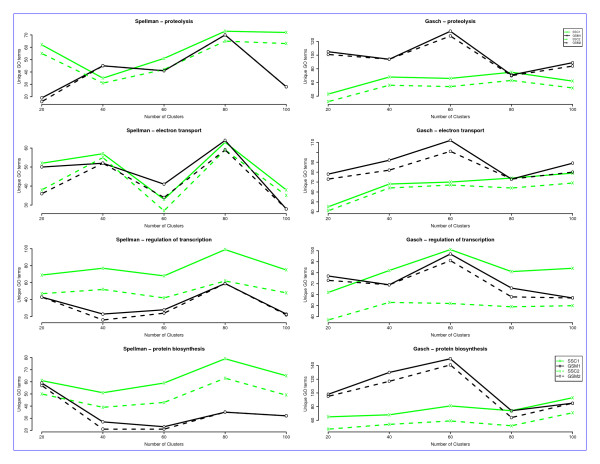
**Comparison of unique GO terms found using gene signatures versus those found using semi-supervized clustering (SSC) for the Spellman and Gasch datasets**. For the semi-supervized clustering (SSC), the landmark genes were considered as 'must-link' constraints. *SSC1 *denotes the number of unique GO terms found by using landmark genes as constraints in SSC. *GSM1 *denotes the number of unique GO terms found by using the gene signature model. *SSC2 *denotes the number of unique GO terms found for SSC if we remove the largest cluster (containing all the landmark genes) from analysis. *GSM2 *denotes the number of unique GO terms found using the gene signature model if we remove the largest cluster from analysis. The results for other landmarks are shown in Figure 3 in Additional File 2.

**Figure 5:**
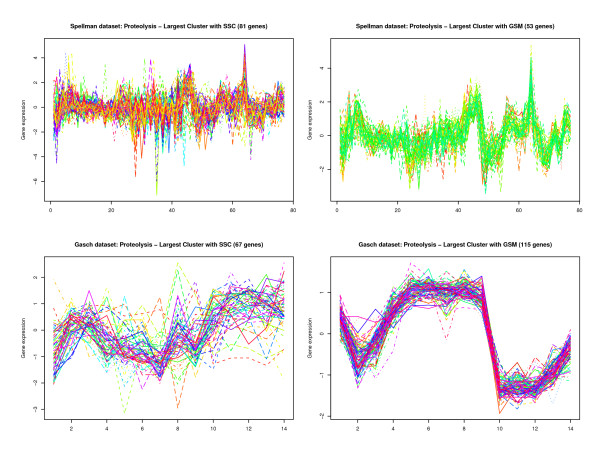
Comparison of gene expression patterns in the largest cluster of semi-supervized clustering (SSC) versus the gene signature model (GSM) for the Gasch dataset using landmark genes associated with 'proteolysis'.

Finally, in order to validate the effectiveness of our approach, we compared our model, using tight clustering with gene signatures (GSM), to an existing semi-supervized clustering (SSC) model. For the SSC, the landmark genes were considered as 'must-link' constraints. All the landmark genes were thus clustered together in one cluster using the SSC. We then compared our model to the SSC by comparing the number of unique GO terms found for each set of landmark genes. We used the Spellman and the Gasch datasets for these experiments. The results of this comparison are shown in Figure 4. These indicate that in general, our model does better for the Gasch dataset while the SSC model does better for the Spellman dataset. The two models may be able to exploit different aspects of the underlying gene expression data. Even for the same set of landmark genes, one model may do better in one dataset than in the other. This is exemplified in the case of 'protein biosynthesis' where the SSC model does better in the Spellman dataset, whereas our model does better in the Gasch dataset (Figure 4). One difference between the two models is that the SSC forces the landmark genes in one cluster. This could lead to a large, less compact cluster, especially in cases where there are a large number of landmark genes with varied expression patterns. For example, for the Spellman dataset (Figure 5), the gene expression pattern of the landmark genes correlates well and the SSC model performs better, whereas for the Gasch dataset (Figure 5) the gene expression pattern of the landmark genes does not correlate well, and the GSM performs better.

### Discussion

These results indicate that clusters using gene signatures have biological significance, and that many of these gene associations are not found using clustering on the original microarray expression datasets. Each set of landmark genes carries the potential of defining its own set of clusters from the same dataset. To study this more closely, we examined several pairs of biological processes, i.e., the biological process that was used for selecting the landmark genes and its corresponding common *unique GO terms *found across datasets. For the Spellman and Gasch datasets, we analyze two of these biological processes (proteolysis and electron transport) and some of their common *unique GO terms*. These are listed in Table 4.

**Table 4:** Some examples of biological processes used to select landmark genes and the common unique GO terms found across the Spellman and Gasch datasets

*Biological Process*	*Common Unique GO terms*
Proteolysis	transcription
	transcription, DNA-dependent
	phosphorylation
	energy reserve metabolism
	microtubule-based process
	sporulation
	sporulation (sensu Fungi)
	cellular lipid metabolism
	regulation of transcription
	reproductive sporulation
	ribosomal large subunit export from nucleus
	regulation of transcription, DNA-dependent
Electron Transport	oxidative phosphorylation
	ATP synthesis coupled electron transport
	ATP synthesis coupled electron transport (sensu Eukaryota)
	cellular respiration
	DNA strand elongation
	phosphorylation
	phosphorus metabolism
	phosphate metabolism
	aerobic respiration

#### Proteolysis and Transcription

The connection between proteolysis and transcription has been well established. Proteolysis has been known to regulate transcription [25,26]. Interaction between the two processes is important for gene control and signaling pathways [27], and for the regulation of the cell cycle [28].

#### Proteolysis and Phosphorylation

The two processes interweave and interact with each other resulting in chromosome replication and segregation in budding yeast [29]. The two processes have also been linked to the Cdc28 protein kinase complex and other proteins involved in the budding yeast [30]. Recently it was reported that the human homolog of Mcm10 (a protein in yeast involved in DNA replication) is also regulated by proteolysis and phosphorylation during the cell cycle [31]. One article explores how the signaling molecule Hedgehog prevents the proteolyis (by phosphorylation) of Cubitus interruptus (Ci-155) transcriptional activator [32] and another touches on how phosphorylation-induced proteolysis eliminates unwanted by-products of protein kinases [33].

#### Electron Transport and Oxidative Phosphorylation

The relationship between these two processes has been studied across organisms. The inhibitory effects of Salicylic Acid on both the mitochondrial functions were presented in [34]. Salicylic acid inhibited mitochondrial electron transport which in turn inhibits oxidative phosphorylation. A recent article has studied the neurological diseases in humans and found that they may be caused by a defective electron transport system and its effect on oxidative phosphorylation [35]. Many other papers have also studied the relationship between these processes [36-39].

#### Electron Transport and ATP Synthesis

The relationship between these two processes has also been well studied. Allakhverdiev [40] studied the role of these two interlinked processes on photodamage and repair in Synechocystis. Electron transport is also tightly coupled to ATP synthesis in chloroplasts [41]. The effect of the two processes on the frequencies and harmonics of yeast Saccharomyces cerevisiae were studied in [42]. Faxen [43] and Belevich [44] study the mechanics of the intermediate steps between Electron transport and energy requiring processes like ATP synthesis.

We chose the Biological Process Ontology to select the landmark genes. Nevertheless, other sources that list genes belonging to a particular process or function can also be used. The biologist should also be able to define their own set of landmark genes and use these as the co-ordinates for projection.

We showed that clustering on gene signatures using different sets of landmark genes creates new sets of clusters that are different from the clusters obtained from the original microarray data. Genes in these new clusters reveal biological insights that were not present in the clustering of the original microarray data. We also showed that the new clusters are associated with biological terms that have some ties with the genes used for landmark selection.

## Conclusion

We have used the SigCalc algorithm to project the microarray data onto a subspace whose co-ordinates are genes (called landmark genes), known to belong to a Biological Process. By changing the biological process and thus the landmark genes, we can change this subspace. We have shown how clustering on this subspace reveals new, biologically meaningful clusters which were not evident in the clusters generated by conventional methods. Each unique choice of a biological process would result in a unique subspace and a new set of clusters, enabling biologists to have more than one interpretation of the dataset. We have used three datasets to show that many of these *unique GO terms *are common across datasets. We have compared our model to an existing model, semi-supervized clustering, and shown that it compares favorably to existing models exploiting some prior knowledge of the data. We have done a literature survey and find strong evidence to support a link between the biological process used to select the landmark genes and the newly found *unique GO terms *that are common across the datasets.

## Methods

### Datasets

We use three yeast Saccharomyces cerevisiae datasets in our experiments. First, we use the cell cycle dataset of Spellman [45] available in R [46], comprising of 5624 genes and 77 samples. Second, we use the diauxic shift dataset of DeRisi [47] comprising of 6066 genes and 7 samples, and third the heat shock dataset of Gasch [48] comprising of 6097 genes and 14 samples. We applied a filter based on variation in gene expression, to focus our computations on informative genes across the samples. We selected genes that had a standard deviation greater than 0.35, and selected only those genes that were annotated in the biological process ontology of GO. The reduced datasets had 2288 genes for Spellman, 2794 genes for DeRisi and 4508 genes for Gasch. We then normalized them to a mean of zero and a standard deviation of one.

### SigCalc

**Figure 6:**
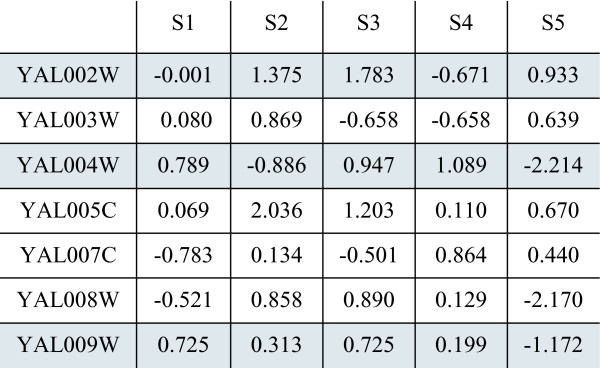
**Microarray expression data matrix**. The selected landmark genes are highlighted.

**Figure 7:**
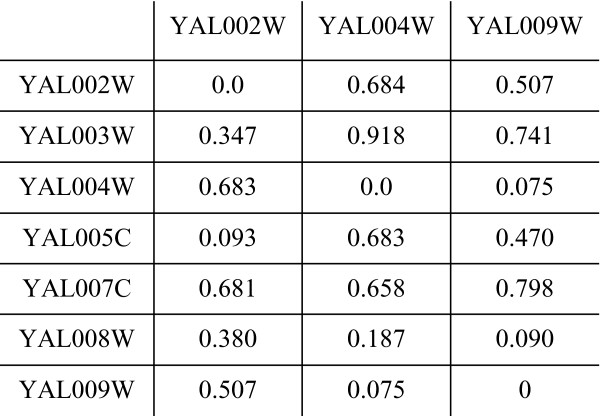
**Gene signatures derived from microarray data using SigCalc**. Gene signature matrix, where each row represents a gene signature.

We introduced the concept of gene signatures in our previous work [49] where it was used as a basis for biological data integration. We formally define the signature calculation algorithm, **SigCalc**, in this subsection. Let *M *represent the microarray table consisting of *n *genes and *m *samples. SigCalc takes as input a microarray table *M *and a biological process. Using Gene Ontology, we find all the GO terms associated with the chosen biological process, and then find all genes associated with these GO terms. These genes are called *landmark genes*. For example, in yeast, the biological process "Protein Folding" is associated with several genes: YHR189W, YCR024C, YMR097C, etc. The algorithm for calculating the gene signatures, given a biological process, is shown in Algorithm 1 [see Appendix]. The **SigCalc **algorithm would convert a microarray data matrix (Figure 6) into a gene signature matrix (Figure 7). SigCalc projects the data onto a subspace, in which each coordinate corresponds to a landmark gene. The projected genes are represented as points in a multi-dimensional subspace. If two genes are close to each other in this projected subspace, then these two genes may show similar expression patterns relative to the landmark genes. By varying the set of landmark genes, we are able to vary this subspace.

The **SigCalc **algorithm uses a distance function, *dist*, to measure the similarity between two gene vectors in microarray *M*. A variety of distance metrics such as Euclidean and cosine distances, or some other variants can be used. In our experiments we used the pearson correlation, a popular similarity metric [50] to arrive at the distance. Given two gene vectors $\vec{g_{i}}$ and $\vec{g_{j}}$, the pearson correlation is given by:

$$cor(\vec{g_{i}},\vec{g_{j}})=\frac{covariance(\vec{g_{i}},\vec{g_{j}})}{\sqrt{covariance(\vec{g_{i}},\vec{g_{i}})\times covariance(\vec{g_{j}},\vec{g_{j}})}}$$

To calculate our gene signatures, we define our correlation distance function as:

$$dist=0.5\times (1-cor(\vec{g_{i}},\vec{g_{j}}))$$

The correlation distance thus ranges from zero to one. A distance of zero indicates perfect positive correlation, and a distance of one indicates perfect negative correlation. A value of 0.5 would indicate no correlation between the gene vectors. Given a set of landmark genes *k *and a microarray *M *containing *n *genes and *m *samples, the SigCalc algorithm will return an *n *× *k *matrix, where each row represents a gene signature, as shown in Figure 7.

### Clustering algorithms used

We chose two popular algorithms, tight clustering that is based on k-means clustering and self organizing maps (SOM) [5] to validate our Gene Signature model. The Tight Clustering algorithm [51] is a re-sampling based algorithm, that uses k-means clustering, to return genes that are clustered together consistently upon resampling. Re-sampling based methods have been found to return consistent clusters [52,53]. The Tight Clustering algorithm forms clusters that are stable and tight, and excludes genes from clusters that are 'noisy' and only serve to dilute the cluster. It has been widely used in microarray data clustering [54-57]. SOM is another clustering algorithm we used in our experiments. We use the R [46] implementation of SOM.

### Cluster validation

Cluster results can be validated using external or internal criteria. External criteria are preferred because they provide a source to validate the clusters independent of the underlying datasets. We use the Gene Ontology to provide this external validation. Gene Ontology validates clustering results by comparing the genes in the clusters to genes known to be associated with specific biological functions. A "good" cluster will have a statistically significant over-representation of genes belonging to a specific biological process, as represented by a *GO term*. Our approach shows how the choice of landmark genes results in different sets of clusters, and that each set of clusters is associated with different sets of biological processes (*GO terms*).

### Significant GO terms from clustering microarray data

We partition the microarray data *M *(*n *genes × *m *samples) into *N *clusters (*N *= 100 for results presented). We evaluate the biological significance of each cluster as follows: For a set of genes in a cluster, we evaluate if there are any GO terms that are over-represented than would be expected by chance. We evaluate the probability of a set of genes in a cluster being associated with the same GO term by using the hypergeometric distribution of the genes in the cluster. The probability of a cluster of size *S *containing *x *genes belonging to a particular GO term, given that the reference dataset of *N *genes has a total of *A *genes belonging to that particular GO term is:

$$Pr\{X=x|N,A,S\}=\frac{\left(\begin{array}{c} A\\ x \end{array}\right)\left(\begin{array}{c} N-A\\ S-x \end{array}\right)}{\left(\begin{array}{c} N\\ S \end{array}\right)}$$

where *X *is a random variable representing the number of genes in a cluster, that are associated with a particular GO term [58]. A cluster is considered to contain a significant GO term only if it has more than two genes associated with a specific GO term, and has a p-value less than 0.01. We used the *GOstat *package [23] for the hypergeometric test to find the set of statistically significant GO terms.

**Figure 8:**
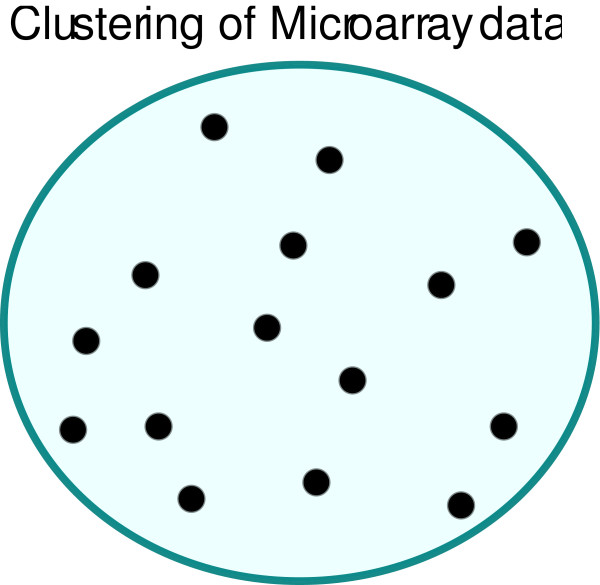
**Significant GO terms in microarray data**. The dots indicate Significant GO terms found by performing clustering on microarray data (i.e., *original GO terms*).

The set of significant GO terms for the original microarray clusters is the union of the significant GO terms for all of the clusters. This set of GO terms will be called the *Original GO terms*, as shown in Figure 8.

### Significant GO terms for a dataset using gene signatures

**Figure 9:**
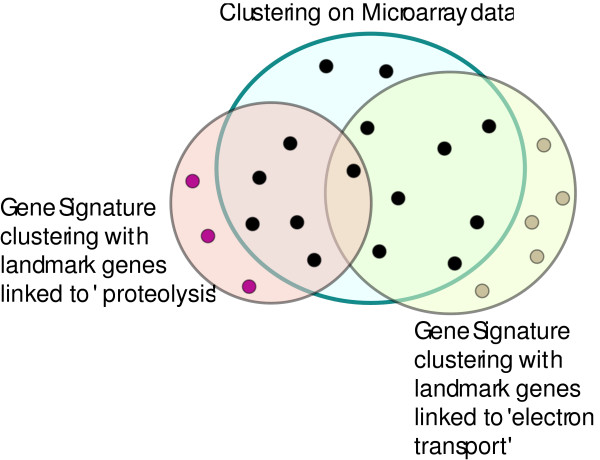
**Significant GO terms in microarray data and in gene signatures**. Shows a comparison of Significant GO terms found by clustering gene signatures (i.e., *landmark GO terms*) with the *original GO terms*.

We build the gene signature matrix, for a selected biological process, by using *SigCalc *as given in Algorithm 1 [see Appendix]. Next, we partition the *n *× *k *gene signature matrix into *N *clusters (*N *= 100, i.e., the same number of clusters that were used for clustering the original microarray data). All other parameters for the clustering algorithm were kept the same as were used to cluster the original microarray data, as described in the previous section. This clustering of Gene signatures will be termed as *Gene Signature Clustering*. The set of significant GO terms from the clusters is derived using the hypergeometric distribution in the same way as described in the previous section. This set of significant GO terms obtained by clustering gene signatures, associated with a set of landmark genes, will be called *landmark GO terms*, as shown in Figure 9. The set of significant GO terms that are present in both the *landmark GO terms *and the *original GO terms *are called *overlapping GO terms*, and the set of significant GO terms that are present in the *landmark GO terms *but not in the *original GO terms *are called *unique GO terms*.

### Unique GO terms common across datasets

Next, we determined if there were *unique GO terms *that were common across datasets. To ensure that the two datasets were comparable, we selected only those genes that were common to both datasets. For example, when comparing the Spellman (2288 genes) and Gasch (4508 genes) datasets, there were 2038 genes that were common to both datasets. So for this comparison, the Spellman dataset comprised 2038 genes × 77 samples, and the Gasch dataset comprised 2038 genes × 14 samples. This also ensured that, for a biological process, the same set of genes would be picked as landmarks for both datasets. For each dataset, we found the *unique GO terms *for a set of landmark genes, and then compared the two sets to determine which *unique GO terms *were common across datasets.

## Source code availability and requirements

*Project name*: Landmark gene-guided clustering.

*Project home page*: 

*Operating system*: Windows

*Programming languages*: R (download at ). All R packages for Gene Ontology can be downlaoded at Bioconductor .

*Licence*: The R source code is freely available under the GPL license. The source code can be obtained as as additional material (see Additional file 1). This source code is provided only for academic use. By using the code, the user agrees to cite the main paper if results obtained from this code are used in the manuscript.

## Competing interests

The author(s) declare that they have no competing interests.

## Authors' contributions

PC, JK and JY contributed to the algorithm design, implementation and systematic analysis of the framework. HC, HK and ML contributed to the biological validation of the findings obtained using the framework. All authors read and approved the final manuscript.

## Appendix

**Input**: Microarray table *M *(*n *genes × *m *samples), and a Biological Process in Gene Ontology (*GO*), *X*.

**Output**: Set of gene signatures *S *= $\vec{sig}$(*g*_1_), ..., $\vec{sig}$(*g*_*n*_).

List all the genes linked to *X *in Gene Ontology. This set of *k *genes are the landmarks and will be represented by *L *= {*l*_1_, ..., *l*_*k*_}.

**foreach ***gene g*_*i *_*in M ***do**

   **foreach ***gene l*_*j *_*in L ***do**

      *d*_*j *_← *dist*($\vec{g_{i}}$, $\vec{l_{j}}$)

   **end**

   $\vec{sig}$(*g*_*i*_) ← [*d*_1_,*d*_2_, ..., *d*_*k*_]

end

**Algorithm 1**: **SigCalc**: Signature Computation Algorithm.

## Supplementary Material

Additional file 1 R source code file Click here for file

Additional file 2 Supplementary Material for 'Microarray data mining using landmark gene-guided clustering' Click here for file
