# Supplementary material for: Microarray data mining using landmark gene-guided clustering
Source: BMC Bioinformatics. 2008 Feb 11;9:92. doi: 10.1186/1471-2105-9-92 (PMC2262871; doi:10.1186/1471-2105-9-92)
Supplement: Additional file 2 — Supplementary Material for 'Microarray data mining using landmark gene-guided clustering' [file 1471-2105-9-92-S2.pdf]

Supplementary Material for 'Microarray data mining using landmark gene-guided clustering'

1. Unique GO terms for DeRisi dataset

Biological process used for landmark genes	*Number of Landmark Genes*	*Number of Original GO terms*	*Number of Overlapping GO terms*	*Number of Unique GO terms*
proteolysis	84	155	107	66
electron transport	24	155	114	113
regulation of transcription	138	155	105	113
protein biosynthesis	321	155	103	112
carbohydrate metabolism	137	155	103	104
signal transduction	70	155	107	109
ubiquitin-dependent protein catabolism	66	155	113	70

Table 1: Details of overlaps between significant GO terms found by original clustering of Microarray data, and those found by using gene signature clustering for the *DeRisi* dataset.

2. Calculating p-value

The probability that two sets A and B, having N_A and N_B number of GO terms, share 'x' GO terms is given by:

$$Pr\{X = x|N_A, N_B, N\} = \frac{\binom{N_A}{x}\binom{N-N_A}{N_B-x}}{\sum_{i=x_{min}}^{x_{max}}\binom{N_A}{i}\binom{N-N_A}{N_B-i}}$$

where,
N = total number of GO terms annotated by the gene set,
$N \geq N_A \geq N_B$,
$x_{max} = N_B$ and,

$$x_{min} = \begin{cases} 0 & \text{if } N_A + N_B \leq N, \\ N_A + N_B - N & \text{if } N_A + N_B > N. \end{cases}$$

3. Gene expression vs. gene signatures.

Some other examples of genes that cluster together only when gene signature vectors are used:

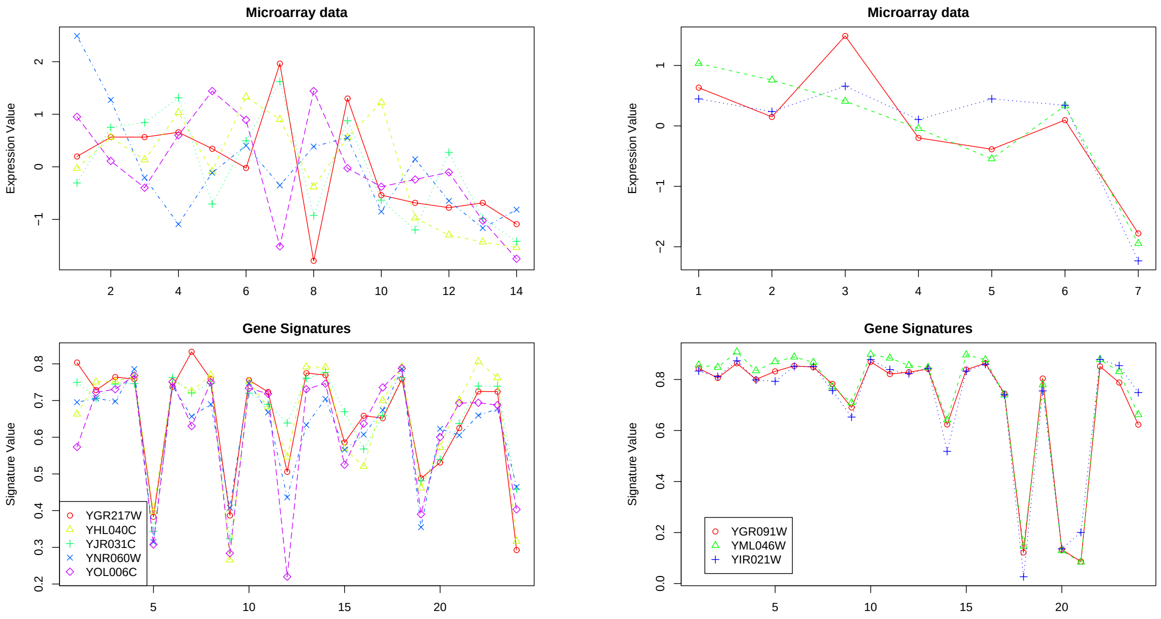

(a) (b)

Figure 1: Comparison of microarray expression data with gene signatures for genes that clustered together using gene signatures. (a)Gasch dataset: Genes associated with *localization* (GO:0051179). (b) DeRisi dataset: Genes associated with *RNA splicing* (GO:0000375).

4. Comparison of accuracy of imputing functional annotation to genes using knn (for varying k) versus using gene signatures.

We do a ten-fold cross validation to impute the function of genes, comparing the accuracies for both knn and gene signatures. For each gene in the test fold, we impute the function of the gene using knn. We use majority voting of the k nearest neighbors to assign a function to the gene. Similarly, for tight clustering with gene signatures, we assign the function to the gene based on the majority vote of all the other genes in the same cluster.

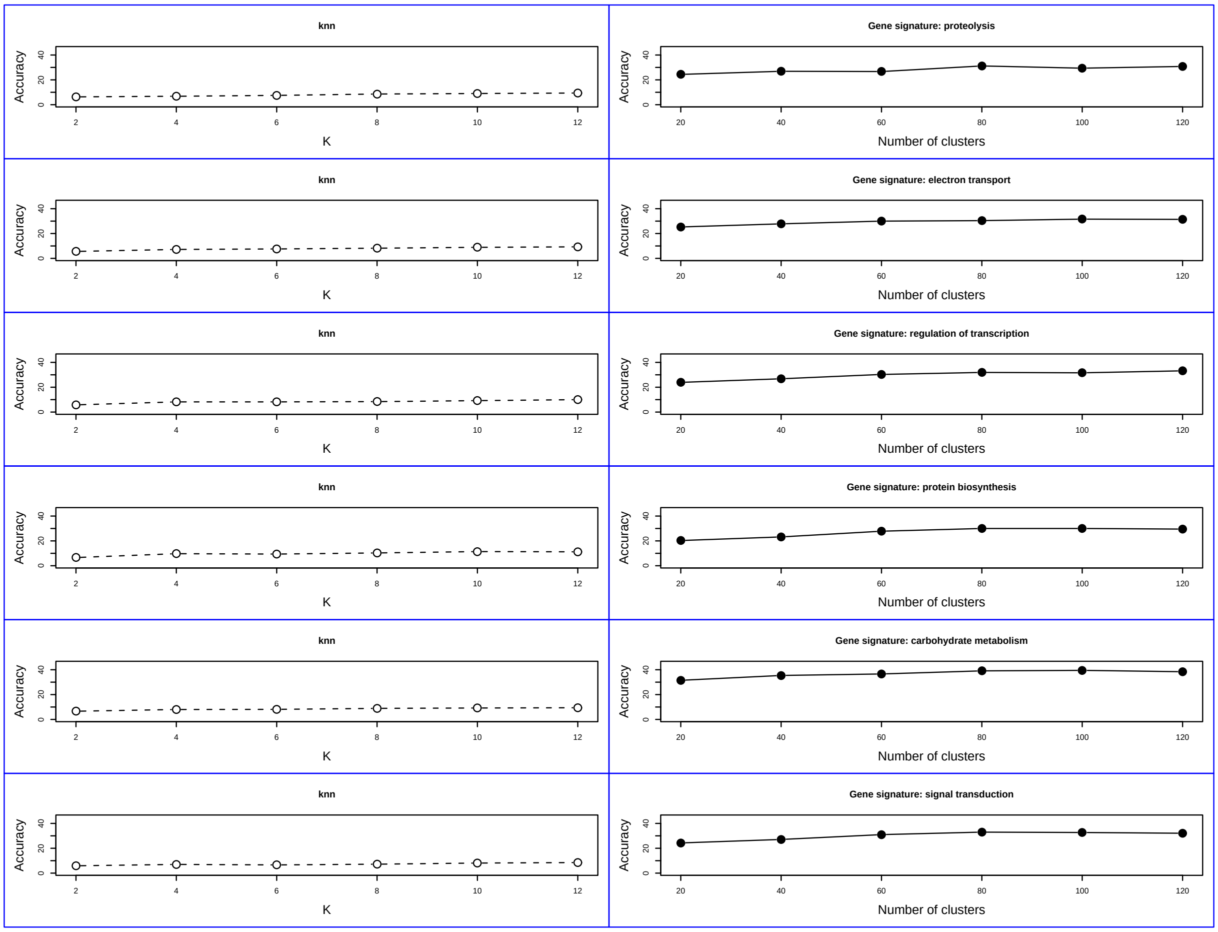

Figure 2: Comparison of accuracy of imputing functional annotation to genes using knn (for varying k) versus using gene signatures.

5. Comparison of unique GO terms found using gene signatures versus those found using semi-supervized clustering for the Spellman and Gasch datasets.

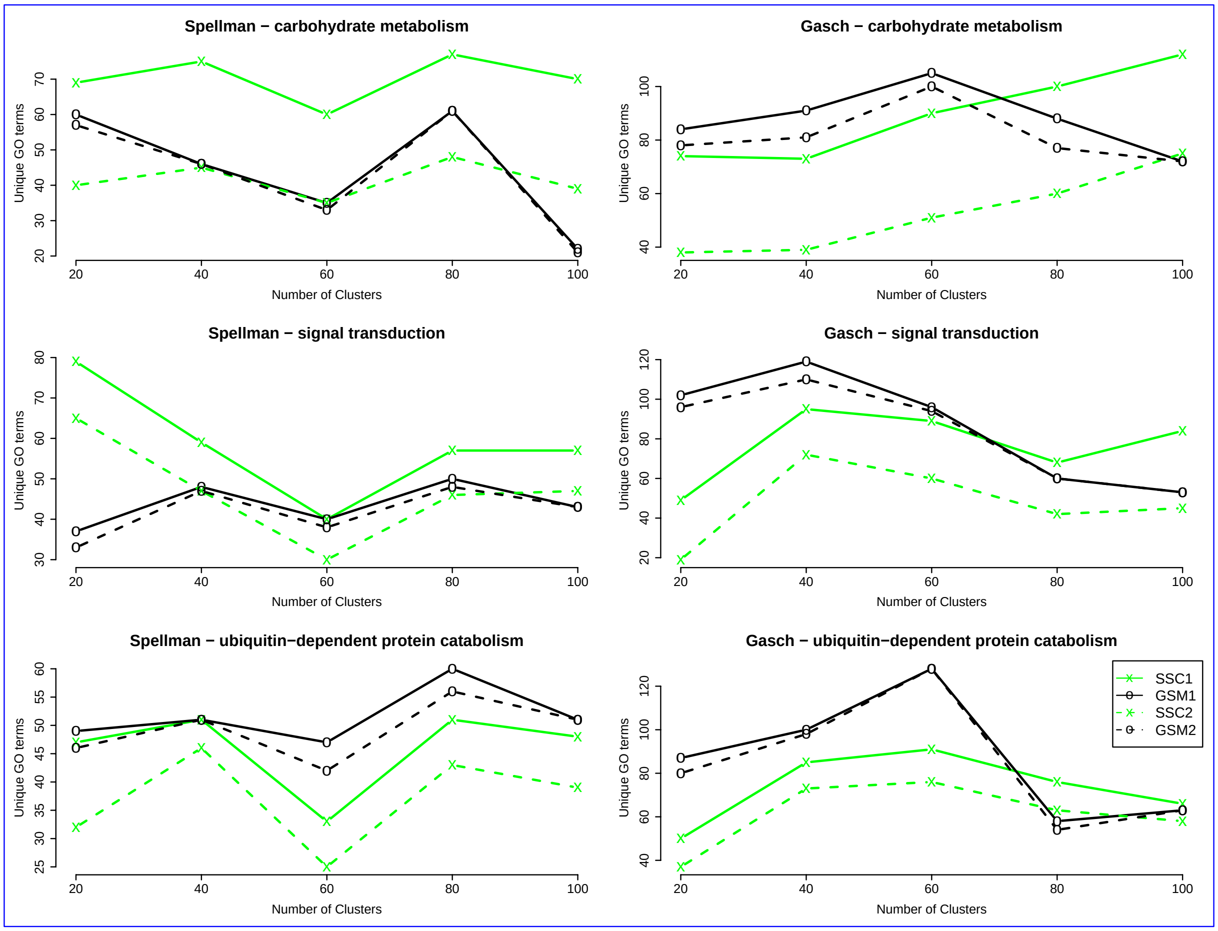

Figure 3: Comparison of unique GO terms found using gene signatures versus those found using semi-supervized clustering (SSC) for the Spellman and Gasch datasets. For the semi-supervized clustering, the landmark genes were considered as 'must-link' constraints. *SSC1* denotes the number of unique GO terms found by using landmark genes as constraints in SSC. *GSM1* denotes the number of unique GO terms found by using the gene signature model. *SSC2* denotes the number of unique GO terms found for SSC if we remove the largest cluster (containing all the landmark genes) from analysis. *GSM2* denotes the number of unique GO terms found using the gene signature model if we remove the largest cluster from analysis.
